# Genotype-Based Bayesian Analysis of Gene-Environment Interactions with Multiple Genetic Markers and Misclassification in Environmental Factors

**DOI:** 10.1155/2012/151259

**Published:** 2012

**Authors:** Iryna Lobach, Ruzong Fan

**Affiliations:** 1 Department of Population Health, Division of Biostatistics, School of Medicine, New York University, New York, NY 10016, USA; 2 Biostatistics and Bioinformatics Branch, Division of Epidemiology, Statistics and Prevention Research, Eunice Kennedy Shriver National Institute of Child Health and Human Development, National Institutes of Health, Rockville, MD 20852, USA

## Abstract

A key component to understanding etiology of complex diseases, such as cancer, diabetes, alcohol dependence, is to investigate gene-environment interactions. This work is motivated by the following two concerns in the analysis of gene-environment interactions. First, multiple genetic markers in moderate linkage disequilibrium may be involved in susceptibility to a complex disease. Second, environmental factors may be subject to misclassification. We develop a genotype based Bayesian pseudolikelihood approach that accommodates linkage disequilibrium in genetic markers and misclassification in environmental factors. Since our approach is genotype based, it allows the observed genetic information to enter the model directly thus eliminating the need to infer haplotype phase and simplifying computations. Bayesian approach allows shrinking parameter estimates towards prior distribution to improve estimation and inference when environmental factors are subject to misclassification. Simulation experiments demonstrated that our method produced parameter estimates that are nearly unbiased even for small sample sizes. An application of our method is illustrated using a case-control study of interaction between early onset of drinking and genes involved in dopamine pathway.

## 1. Introduction

A key component to prevention and control of complex diseases, such as cancer, hypertension, diabetes, and alcoholism, is to study the independent, cumulative, and interactive effects of genetic and environmental factors. This analysis has the potential to impact the understanding of the role of genetic influences under various environmental exposures, thus providing valuable information to (1) better understand the biological pathways involved in the disease and its progression, thus providing major clues to the underlying causes of alcohol dependence; (2) design personalized interventions targeted to individuals with enhanced vulnerability to the disease (the risk genes may help identify patients at higher risk long before any symptoms occur); (3) gain critical understanding for drug discovery.

This work is motivated by the following two concerns in the analysis of gene-environment interactions. **First**, complex diseases are caused by multiple variants with small-to-moderate effect sizes working in concert [1]. Most of the results of published genome-wide association studies are based on single nucleotide polymorphism (SNP) analysis [2]. This approach may suffer from low power due to a large number of tests and small effect sizes of individual SNPs. Furthermore, the true causal genetic marker is often not genotyped, rather is captured through linkage disequilibrium (LD) with the typed markers. Since each SNP has only partial linkage disequilibrium with the causal SNP, the observed effect size of the typed SNP is lower than the effect size of the causal SNP. In light of this concern, we propose to use a risk function that allows the genetic markers in linkage disequilibrium to enter the model directly [3]. This model eliminates the need to estimate haplotype phase and hence protects against bias due to the uncertainty that may arise due to the haplotype phase ambiguity [4–8]. In addition, the computation burden can be significantly reduced since the proposed approach uses genotype data directly. **Second**, many variables that are of interest to biomedical researchers are subject to misclassification, for example, due to uncertainty associated with a recall or a measurement at an individual level. Misclassification may result in bias and loss of power to detect gene-environment interactions [9]. Oftentimes uncertainty associated with these variables may not be avoided in practice. The loss of power prevents the ability to discover gene-environment interactions in small studies or studies involving analysis of subtypes of complex diseases.

An example of biomedical problem of gene-environment interactions is the analysis of role of age when first got drunk in the etiology of alcohol dependence. The age at which a person gets drunk for the first time may influence genes linked to alcoholism, making the youngest drinkers most susceptible to severe problems [10]. Twin study found that when twins started drinking early (age < 13 years old), genetic factors contributed greatly to risk for alcohol dependence, at rates as high as 90 percent in the youngest drinkers [10]. Some early-onset drinkers do not develop alcohol problems and some late-onset drinkers do, hence it is important to investigate genetic and environmental influences that predispose for or protect against alcohol dependence in these two groups. However, the definition of early age of getting drunk is subject to misclassification due to uncertainty associated with the recall.

In light of these concerns, we develop a Bayesian methodology for analysis of gene-environment interactions in case-controls studies. Estimation and inference are based on a pseudolikelihood function [3, 11, 12]. This pseudolikelihood function offers the following advantages. One is that environmental variables measured exactly are modeled completely nonparametrically. Furthermore, *a priori* information about the probability of disease can be incorporated directly. The pseudolikelihood function exploits gene-environment independence assumption which is a reasonable assumption in many practical applications. If the gene-environment interaction is not significantly present in the population, then the distribution of genotype can be specified within strata defined by an environmental covariate. The proposed analysis is based on a pseudolikelihood function hence conventional Bayesian techniques may not be applied directly. Validity of Bayesian techniques need to be examined when the likelihood function is not a proper likelihood [13]. We followed Monahan and Boos [13] and Lobach et al. [3] to validate our Bayesian approach under this pseudolikelihood function. Our Bayesian approach has the ability to shrink the parameter estimates towards prior and hence reduce variability in parameter estimates. This property is essential when environmental exposure is subject to misclassification, especially in studies with smaller sample sizes, for example, of subtypes of complex disease. On the other hand, if sample size is large enough, estimation and inference can be based on the asymptotic posterior distribution that we derived which will ease the computational burden.

An outline of this paper is as follows. In Section 2 we introduce notation and formally state the problem. In Section 2 we present the Bayesian model under various scenarios. Section 3 describes asymptotic posterior distribution. Section 4 describes simulation experiment. Section 5 describes application of the Bayesian model to the analysis of alcoholism study. Section 6 gives concluding remarks.

## 2. Bayesian Model Based on Pseudolikelihood

### 2.1. Notation and Risk Function

Consider a sample consisting of *n*_0_ controls and *n_d_* cases at disease stage or type *d* = 1, *…*, *K*. Define *D* as the disease status. Following Lobach et al. [11], we pretend that this case-control sample is collected using a simple Bernoulli scheme, where the selection probability of a subject given disease status is proportional to *n_d_/*pr(*D* = *d*), *d* = 0, 1, *…*, *K*. Let *R* = 1 denote the indicator of whether or not a subject is selected into the case-control sample. All participants of the study will have this selection status *R* = 1. The observed genetic data consist of unphased genotypes *G* = (*G*_1_, *…*, *G_I_*) at *I* loci. Let *Q*(*G; θ*) be a model describing Hardy-Weinberg equilibrium (HWE).

Let (*T*, *Z*) denote all nongenetic variables of interest. Suppose *T* is the set of factors subject to misclassification, and *Z* is the set of variables observed exactly. We assume that the observed genetic data does not contain any additional information on disease status and the true environmental covariate given the genetic variable of interest. Let *X* denote the error-prone version of *T*. Suppose the misclassification process is defined by the following parametric structure *p*_miss_(*x* | *T*, *G*, *Z*, *D*, *ξ*). This model is general enough to capture differential misclassification. The joint distribution of the environmental factors in the underlying population can be specified in the following form *p_T_*_|_*_Z_*(*t* | *z*, *ξ*)*f_Z_*(*z*). While *T* may be a vector of factors, for simplicity of presentation in what follows we suppose that *T* is a factor.

Given the environmental covariates *T* and *Z*, genotype data *G*, the risk of disease in the underlying population is given by the following polytomous logistic model:

(2.1) $$\text{pr}(D=k\geq 1|G,T,Z)=\frac{\exp\;\{\beta_{k0}+m_{k}(G,T,Z;\beta )\}}{1+\sum_{j=1}^{K}\exp\;\{\beta_{j0}+m_{j}(G,T,Z;\beta )\}},$$

where *m*(●)is a function of known form parameterizing the risk of disease in terms of parameters *β*. For the *i*th marker, denote the two alleles by *M_i_* and *m_i_*, with frequencies *P*_*M*_*i*__ and *P*_*m*_*i*__, respectively. Following Lobach et al. [3], we define the following dummy variables and two risk models: genotype effect model and additive effect model.

Define the following dummy variables:

(2.2) $$A_{i}=\left\{ \begin{array}{ll} 1, & \text{if}\;G_{i}=M_{i}M_{i},\\ 0, & \text{if}\;G_{i}=M_{i}m_{i},\\ -1, & \text{if}\;G_{i}=m_{i}m_{i}, \end{array}\right.\;\;\;B_{i}=\left\{ \begin{array}{ll} -P_{m_{i}}^{2}, & \text{if}\;G_{i}=M_{i}M_{i},\\ P_{M_{i}}P_{m_{i}}, & \text{if}\;G_{i}=M_{i}m_{i},\\ -P_{M_{i}}^{2}, & \text{if}\;G_{i}=m_{i}m_{i}. \end{array}\right.$$

Notice that *A_i_* + 1 is the number of allele *M_i_* at the *i*th marker, and hence *A_i_* can be used to model the allele or additive effect of *M_i_*. Let pr(*g; θ*) be a parametric form of the joint distribution of the observed genetic markers. In the following, we provide two examples of function *m_k_*(·) using the genotype information **G** = (*G*_1_, *G*_2_, *…*, *G_I_*).

#### 2.1.1. Genotype Effect Model (GEM)

The following specification of the risk function incorporates both additive and dominance effects of genotype, as well as the multiplicative gene-environment interactions

(2.3) $$\begin{array}{l} m_{k}(G,T,Z;\beta )=m_{k}(A,ℬ,T,Z,\beta )=T\beta_{kT}+Z\beta_{kZ}+\sum_{i=1}^{I}A_{t}\beta_{\mathrm{kAi}}\\ +\sum_{i=1}^{I}TA_{i}\beta_{\mathrm{kATi}}+\sum_{i=1}^{I}ZA_{i}\beta_{\mathrm{kAZi}}+\sum_{i=1}^{I}B_{i}\beta_{\mathrm{kDi}}+\sum_{i=1}^{I}TB_{i}\beta_{\mathrm{kDTi}}+\sum_{i=1}^{I}ZB_{i}\beta_{\mathrm{kDZi}}. \end{array}$$

In this formulation, the regression coefficients *β_kAi_* and *β_kDi_* model risk due to the additive and dominance effect, respectively [14, 15]. The remaining terms capture the multiplicative gene-environmental interaction.

#### 2.1.2. Additive Effect Model (AEM)

Suppose that the dominance effect is not significantly present in the model (2.3). In this situation, the risk function takes the following form:

(2.4) $$m_{k}(G,T,Z;\beta )=m_{k}(A,T,Z;\beta )=T\beta_{kT}+Z\beta_{kZ}+\sum_{i=1}^{I}A_{i}\beta_{\mathrm{kAi}}+\sum_{i=1}^{I}TA_{i}\beta_{\mathrm{kATi}}+\sum_{i=1}^{I}ZA_{i}\beta_{\mathrm{kAZi}}.$$

### 2.2. Pseudolikelihood

Let us denote *κ_k_* = *β_k_*_0_ + log(*n_k_*/*n*_0_)−log(*π_k_*/*π*_0_), *k* = 1, 2, *…*, *K*, and *κ̃* = (*κ*_1_, *…*, *κ_K_*)*^T^*. In addition, let *β̃*_0_ = (*β*_10_, *…*, *β_K_*_0_)*^T^*, $\Omega ={\left({\tilde{\beta }}_{0}^{T},\beta^{T},\Theta^{T},{\tilde{\kappa }}^{T}\right)}^{T}$, 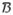 = (Ω*^T^, η^T^*)*^T^*, and *υ* = (*η^T^, ξ^T^*)*^T^*. Define

(2.5) $$S(k,g,t,z;\Omega )=\frac{\exp\;[1_{\left(k\geq 1\right)}(k)\;\{\kappa_{k}+m_{k}(g,t,z;\beta )\}]}{1+\sum_{j=1}^{K}\exp\;\{\beta_{j0}+m_{j}(g,t,z;\beta )\}}\text{pr}(g;\Theta ).$$

We assume that *G* and (*X, Z*) are independently distributed in the underlying population. Only changes in notation are needed to model genotype and environment within strata thus relaxing gene-environment independence assumption. An example of gene-environment dependence is polymorphisms in nicotine metabolism pathway that may regulate the degree of addiction to nicotine, thus creating gene-environment interaction. Furthermore, these polymorphisms may interact with smoking status while being involved in lung cancer [16]. We suppose that the type of genetic covariate measured does not depend on the individual’s true genetic covariate, given disease status, environmental covariates and the measured genetic information. Furthermore, we suppose that the observed genetic variable does not contain any additional information on disease status and true environmental covariate given the genetic variable of interest.

Similarly to Lobach et al. [11], we propose to use the following pseudolikelihood function in place of the likelihood function to estimate the parameters:

(2.6) $$\begin{array}{l} L_{\text{Pseudo}}(k,g,x,z;\Omega ,\eta ,\xi )\equiv \text{pr}(D=k,\;G=g,\;X=x|Z=z,\;R=1)\\ =\frac{\sum_{t^{*}}S(k,g,t^{*},z;\Omega )p_{\text{miss}}(x|k,g,t^{*},z;\xi )f_{T}(t|z;\eta )}{\sum_{k^{*}=0}^{K}\sum_{t^{*}}\sum_{g\in G}\int S(k^{*},g,t^{*},z;\Omega )f_{T}(t^{*}|z;\eta )}, \end{array}$$

where 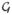 is the set of all possible genotypes in the population. Lobach et al. [12] proved that maximization of *L*_Pseudo_, although not the actual retrospective likelihood for case-control data, leads to consistent and asymptotically normal parameter estimates. Observe that conditioning on *Z* in *L*_Pseudo_ allows it to be free of the nonparametric density function *f_Z_*(*z*), thus avoiding the difficulty of estimating potentially high-dimensional nuisance parameters.

### 2.3. Bayesian Analysis Based on Pseudolikelihood

Since in our setting the retrospectively collected data is analyzed as if they were coming from a random sample, function (2.6) is not a real likelihood function and hence the traditional Bayesian analysis is not technically correct. Conventional approaches to validity of posterior probability statements follow from the definition of the likelihood as the joint density of observations.

For simplicity of presentation we introduce new notation for this section only.

Monahan and Boos [13] introduced a definition based on coverage of posterior sets that are constructed to contain the correct probability of including a parameter *τ*, if the underlying distribution of *τ* is the prior *p*(*τ*) and the model *f*(*Y* | *τ*) of data *Y* is correct. This approach has been used in gene-environment interaction setting [3]. For example, in the one-dimensional case, the natural posterior coverage set functions are the one-sided intervals $I_{\alpha }^{*}=R_{\alpha }(Y)=(-\infty ,\tau_{\alpha }^{*})$, where $\tau_{\alpha }^{*}$ is *α*-percentile of the posterior *f*(*Y* | *τ*). Validity for such a posterior means that all these intervals $I_{\alpha }^{*}$ have the correct coverage *α*. In practice, it is often challenging to verify the required probability analytically. Monahan and Boos [13] proposed a convenient numerical method. Briefly, define *τ_k_*, *k* = 1*, …, m* to be a sample generated independently from a continuous prior *p*(*τ*). For each *τ_k_*, let *Y^k^* denote a value generated from *f*(*Y* | *τ_k_*). In addition, for each *k*, define *H_k_* to be a variable in the following form:

(2.7) $$H_{k}=\int_{-\infty }^{\tau_{k}}f\left(\tau |Y^{k}\right)d\tau .$$

This corresponds to posterior coverage set functions of the form (−∞, $\tau_{\alpha }^{k}$), where $\tau_{\alpha }^{k}$ is the *α*th percentile point of posterior density *f*(*τ* | *Y^k^*). Monahan and Boos [13] argued that if the distribution of *H_k_* fails to follow the uniform distribution for any prior, then the likelihood function cannot be a coverage proper Bayesian likelihood.

We propose to use the methodology described above to validate the likelihood function and apply conventional MCMC techniques to estimate parameters. We note that the method developed by Monahan and Boos is devised to invalidate a pseudolikelihood. Therefore to validate a pseudolikelihood, we propose to consider a comprehensive set of scenarios to examine coverage probabilities of posterior sets, and if these scenarios fail to invalidate a pseudolikelihood, we suppose that it is valid.

### 2.4. Fully Bayesian Model

We consider the case when the environmental covariates (*T, X)*, genetic variant *G*, and disease status *D* are binary. Let pr(*G* = 1) = *θ*, pr(*T* = 1) = *η*. For simplicity of presentation, consider an additive model. Define the vector of risk parameters 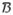 = (*β_t_, β_A_, β_B_, β_tA_, β_tB_*)*^T^*. Consider a multiplicative interaction and let *m*(*t, g,* 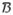) = *β_t_t* + *β_A_A* + *β_tA_tA* + *β_B_B* + *β_tB_tB*. Make the following definition:

(2.8) $$S(d,g,t,ℬ,\theta )=\frac{\exp\;[I_{\left(d\geq 1\right)}(d)\;\{\kappa_{d}+m(t,g,ℬ)\}]}{1+\exp\;\{\beta_{0}+m(t,g,ℬ)\}}\theta^{g}{\left(1-\theta \right)}^{1-g}.$$

If *X* is an observed environmental covariate prone to misclassification, denote the misclassification probabilities as pr(*X* = 1 | *T* = 0) = *ξ*_1_ and pr(*X* = 0 | *T* = 1) = *ξ*_0_. Hence, the distribution of misclassification process is *f*_mem_(*x* | *t, ξ*_0_*, ξ*_1_) = {*xξ*_1_ + (1 − *x*)(1 − *ξ*_1_)}(1 − *t*) + {*x*(1 − *ξ*_0_) + (1 − *x*) *ξ*_0_}*t*.

On the risk parameters, we impose a normal prior with mean 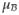 and covariance matrix 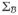.

Similarly to the appendix in Fan and Xiong [14] and Lobach et al. [3], the following expectations, variances, and covariances can be derived. *E*(*A_i_*) = *P*_*M*_*i*__ − *P*_*m*_*i*__, *E*(*B_i_*) = 0, Var(*A_i_*) = 2*P*_*M*_*i*__*P*_*m*_*i*__, $\text{Var}(B_{i})=P_{M_{i}}^{2}P_{m_{i}}^{2}$, Cov (*A_i_, A_j_*) = 2Δ_*M*_*i*_*M*_*j*__, $\text{Var}(B_{i},B_{j})=\Delta_{M_{i}M_{j}}^{2}$, *i*≠*j*. And Cov(*A_i_, B_i_*) = 0 for all *i* and *j*;

(2.9) $$V_{A}=2\left(\begin{array}{cccc} P_{M_{1}}P_{m_{1}} & \Delta_{M_{1}M_{2}} & \cdots & \Delta_{M_{1}M_{I}}\\ \Delta_{M_{1}M_{2}} & P_{M_{2}}P_{m_{2}} & \cdots & \Delta_{M_{2}M_{I}}\\ \vdots & \vdots & \cdots & \vdots \\ \Delta_{M_{1}M_{I}} & \Delta_{M_{2}M_{I}} & \cdots & P_{M_{I}}P_{m_{I}} \end{array}\right),\;\;\;V_{D}=\left(\begin{array}{cccc} P_{M_{1}}^{2}P_{m_{1}}^{2} & \Delta_{M_{1}M_{2}}^{2} & \cdots & \Delta_{M_{1}M_{I}}^{2}\\ \Delta_{M_{1}M_{2}}^{2} & P_{M_{2}}^{2}P_{m_{2}}^{2} & \cdots & \Delta_{M_{2}M_{I}}^{2}\\ \vdots & \vdots & \cdots & \vdots \\ \Delta_{M_{1}M_{I}}^{2} & \Delta_{M_{2}M_{I}}^{2} & \cdots & P_{M_{I}}^{2}P_{m_{I}}^{2} \end{array}\right).$$

Define 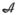 = (*A*_1_*, …, A_I_*) and 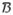 = (*B*_1_*, …, B_I_*). Let **O_I_** be a *I* × *I* matrix with zero elements. Based on the expectations and covariances described above, we have $\text{Cov}(A,ℬ)=\left(\begin{array}{cc} V_{A} & O_{I}\\ O_{I} & V_{D} \end{array}\right)$.

In the case when misclassification is large, the sampling distribution of risk parameter estimates is likely to be skewed [11, 17]. However, because the shape of the normal distribution is symmetric, this prior is likely to bring the sampling distribution of the risk parameter estimates closer to normal. For the frequency parameters *η* and *θ*, we use noninformative uniform (0,1) priors. In this setting, the prior information imposed on *θ* is noninformative. If *a priori* information is available about the genotype frequencies, it can be specified using a corresponding distribution or HWE.

Then, the joint posterior distribution for the model unknowns is proportional to

(2.10) $$\begin{array}{l} \prod_{i=1}^{n}\frac{\sum_{t^{*}=0}^{1}S(d_{i},g_{i},t^{*},ℬ,\theta )p_{\text{miss}}(x_{i}|t^{*},\xi_{0},\xi_{1})\;\eta^{t^{*}}\;{\left(1-\eta \right)}^{1-t^{*}}}{\sum_{t^{*}=0}^{1}\sum_{d=0}^{1}\sum_{g=0}^{1}S(d_{i},g_{i},t^{*},ℬ,\theta )\;\eta^{t^{*}}\;{\left(1-\eta \right)}^{1-t^{*}}}\\ \times {|\sum_{ℬ}|}^{-1/2}\exp\;\left\{-\frac{1}{2}{\left(ℬ-\mu_{ℬ}\right)}^{T}\sum_{ℬ}^{-1}(ℬ-\mu_{ℬ})\right\}\times I_{\left(0,1\right)}(\eta )\times I_{\left(0,1\right)}(\theta ). \end{array}$$

Note that in this formulation, we specify a known misclassification process. We recommend performing sensitivity analysis to see whether parameter estimates change when misclassification probabilities are specified slightly differently. Furthermore, we recommend conservative setting when LD is set to be zero as *a priori*.

## 3. Asymptotic Posterior Distribution

We now consider properties of an asymptotic posterior distribution based on the pseudolikelihood (2.6). MCMC techniques can be computationally challenging. Knowing the form of an asymptotic posterior distribution would ease the computational burden.

For simplicity, we suppose that the parameter *ξ* that controls misclassification error distribution is known, although this is not required. Denote Θ_0_ and Θ̂*_n_* to be values that maximize prior and pseudolikelihood, respectively. Let Ψ(*d, g, x, z,* Θ*, ξ*) be the derivative of log{*L_i_*(*d, g, x, z,* Θ*,ξ*)} with respect to Θ and

(3.1) $$\Lambda =\sum_{d}\frac{n_{d}}{n}E\{\Psi (D,G,X,Z,\Omega ,\eta ,\xi )|D=d\}\times E{\left\{\Psi (D,G,X,Z,\Omega ,\eta ,\xi )|D=d\right\}}^{T}.$$

Furthermore, if *p*(Θ) is the prior distribution of the vector of parameters, define *l*(Θ) to be the derivative of log{*p*(Θ)} with respect to Θ. Then define

(3.2) $$L_{n}(\Theta ,\xi )=\sum_{i=1}^{n}\Psi (D_{i},G_{i},X_{i},Z_{i},\Theta ,\xi )$$

and matrices

(3.3) $$I(\Theta )=-E\;\left[\frac{\partial \{L_{n}(\Theta ,\xi )\}}{\partial (\Theta )}\right];\;\;\;J(\Theta )=-E\;\left[\frac{\partial \{l(\Theta )\}}{\partial (\Theta )}\right].$$

Bernardo and Smith [18] showed that under suitable regularity conditions the posterior distribution of vector of parameters Θ̂*_n_* converges to normal 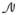
( 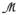, Σ) distribution. Mean vector and covariance matrix can be consistently estimated as follows:

(3.4) $$\begin{array}{c} \hat{M_{n}}={\hat{\sum }}_{n}^{-1}\left\{I\left({\hat{\Theta }}_{n}\right){\hat{\Theta }}_{n}+J(\Theta_{0})\Theta_{0}\right\},\\ {\hat{\sum }}_{n}{\left\{I({\hat{\Theta }}_{n})+J(\Theta_{0})\right\}}^{-1}. \end{array}$$

It can be easily seen that *n*^−1^*∂*{ 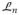
( 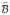
*, ξ*)}/*∂* 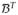 is a consistent estimate of 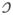
(Θ). Alternatively, if Σ̂ is the sample covariance matrix of the terms Ψ(*D_i_, G_i_, X_i_, Z_i_,* 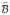, *ξ*), then Σ̂ + Λ̂ consistently estimates 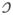
(Θ).

Note that the posterior distribution has precision equal to the sum of precision provided by the observed data and the prior precision matrix. This formulation suggests an approximation, namely, that for large *n*, prior is small compared to the one provided by the observed data. Hence, with a large sample size, one can reduce computational burden by using the asymptotic distribution and using precision provided by the observed data while specifying the posterior distribution.

## 4. Simulation Experiments

We investigated the case of small *n*_0_ = *n*_1_ = 350 and large (*n*_0_ = *n*_1_ = 1,500) sample sizes.

We validated the pseudolikelihood function using methodology described by Monahan and Boss [13] in a few scenarios by varying sample size, effect size, and misclassification probabilities. In 96% of cases that we considered, the Kolmagorov-Smirnov test failed to reject the null hypothesis that the sample of *H_k_* (2.7) comes from the uniform (0,1) distribution at the 0.05 significance level. Hence, we concluded that the pseudolikelihood is valid for subsequent analysis. Hence, we proceeded to estimating parameters.

We implemented Metropolis-Hastings algorithm in the following setting. On the risk parameters 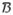, we imposed a normal 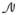
( 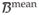, 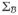) prior, where 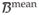 is equal to the pseudo-MLE estimates. To examine sensitivity of the estimates to this specification, we considered a case when 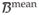 is a vector of zero values. Covariance matrix was specified as a diagonal matrix with diagonal elements equal to 3^2^. Alternatively, we specified the corresponding matrix according to the known structure that is a function of LD. In all of these scenarios, the results we obtained were comparable. Table 1 presents results based on 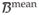 = (0, 0, 0) and covariance matrix with diagonal elements equal to 3^2^.

To examine performance of our approach, we performed two simulation experiments. In the first experiment, we investigated performance of Bayesian method compared to pseudo-MLE. The goal of this experiment was to examine the ability of Bayesian approach to shrink the parameter estimates towards prior when misclassification causes the estimates to have skewed distribution. In the second experiment, we examined performance of the asymptotic posterior distribution.

### Experiment 1

We generated the true environmental variables *T* from a binomial distribution with pr(*T* = 1) = 0.5. The misclassification probabilities are pr(*X* = 0 | *T* = 1) = 0.20 and pr(*X* = 1 | *T* = 0) = 0.25. We simulated three genetic markers in LD corresponding to Δ = 0.03 and *P*_*M*_*i*__ = 0.25. In the study with 1, 500 cases and 1, 500 controls, we generated a binary disease status according to the following logistic model:

(4.1) $$\text{logit}\{\text{pr}(D=1|G,X)\}=\beta_{0}+\beta_{t}t+\sum_{j=1}^{3}\beta_{A_{j}}A_{j}+\sum_{j=1}^{3}\beta_{B_{j}}B_{j}+\sum_{j=1}^{3}\beta_{{AT}_{j}}A_{j}T+\sum_{j=1}^{3}\beta_{{TB}_{j}}B_{j}T.$$

To examine the case when genetic data is missing, we simulated a similar set of 1,500 cases and 1,500 controls with 50% of genetic information missing completely at random. To investigate a smaller study, we simulated 350 cases and 350 controls with the disease status defined by the risk model with all *β*_*B*_*j*__and *β_BTj_* set to 0. Results presented in Tables 1 and 2 illustrate that the proposed Bayesian approach produced parameter estimates that are less variable and less biased. We examine the empirical distribution of parameter estimates based on a small sample and found that it is skewed, which may be due to small sample size and presence of misclassification. We observed this phenomena in our previous work [3, 11]. The Bayesian solution brings the advantage, that is, a symmetric prior can shrink parameter estimates towards normal distribution. Furthermore, we presented performance of the naive approach that ignores existence of misclassification.

### Experiment 2

We examined performance of estimation based on the derived asymptotic posterior in the simulation setup described in Experiment 1 corresponding to *n*_1_ = *n*_2_ = 1, 500. Results presented in Table 3 illustrate that the parameter estimates are nearly unbiased. Moreover, estimated variances of parameter estimates are very close to the observed variability with one exception, namely, *β_x_*. Variability of *β_x_* may be inflated due to the misclassification in environmental exposure.

## 5. Analysis of Alcohol Dependence

The Collaborative Studies on the Genetics of Alcoholism (COGA) is a nine-center nationwide study that was initiated in 1989 and has had as its primary aim the identification of genes that contribute to alcoholism susceptibility and related characteristics [19–21]. COGA is funded through the National Institute on Alcohol Abuse and Alcoholism (NIAAA). The focus of this study is a case-control design of unrelated individuals for a genetic association analysis of addiction. Analyses that include incorporation of important demographic and environmental factors such as age when first got drunk, sex, income, and education into association studies are pursued. Our project involves analysis of 40 SNPs residing in genes involved in dopamine pathways. Specifically, we consider D2 dopamine receptor gene (DRD2) encoding a protein which plays a central role in reward-mediating mesocorticolimbic pathways; a member of the immunoglobulin gene superfamily NCAM1 encoding protein involved in various neural functions; tetratricopeptide repeat domain 12 gene (TTC12); CHRNA3 gene shown to be involved in higher craving after quitting and increased withdrawal symptoms over time. Cases are defined as individuals with DSM-IV alcohol dependence (lifetime). Controls are defined as individuals who have been exposed to alcohol, but have never met lifetime diagnosis for alcohol dependence or dependence on other illicit substances. The sample consists of 50.7% of male and 49.3% female participants; 60% report their race as Caucasian and 40% are non-Caucasian. We categorized age when first got drunk as “Early” if it is less or equal to 13 (EAD = 1, 45.2% of all participants) and people with low income are the ones who make less than 30 K per year (LI = 1, 45% of all participants).

Define *T* to be the true unobserved indicator of early drinking, that is, *T* = 1 corresponds to the early onset of drinking, *T* = 0 to the late onset. Let X be the observed value of the early onset of drinking. Because we do not have external data or internal replicates to estimate misclassification probability, we performed sensitivity analysis for various values of misclassification.

We used the following risk model:

(5.1) $$\text{logit}\{\text{pr}(D=1|G=(A,B),T)\}=\beta_{0}+\beta_{t}T+\beta_{A}A+\beta_{B}B+\beta_{AT}AT+\beta_{BT}BT.$$

The results of sensitivity analysis (not shown) suggest that when pr(*X* = 0 | *T* = 1) is ignored or underestimated, the interaction effect is not significant. The setting corresponds to the case when exposed subjects are defined as nonexposed, thus reducing the association signal. However, the estimation procedure appears to be robust to underestimation of pr(*X* = 1 | *T* = 0). This scenario corresponds to the case when a nonexposed subject is considered to be exposed.

Parameter estimates obtained using our method corresponding to pr(*X* = 0 | *T* = *t*) = 0.25 and pr(*X* = 1 | *T* = 0) = 0.25 are presented in Table 4 demonstrating significant interaction between various genetic markers and early onset of drinking.

## 6. Discussion

Motivated by concerns in the analysis of gene-environment interactions, we proposed a genotype-based Bayesian approach for the analysis of case-control studies when environmental exposure cannot be observed directly and is subject to misclassification. The formulation of risk functions and the estimation procedure are along the lines of our previous work: genotype and additive effect models [14, 15] and pseudolikelihood approach [3, 11, 12]. The risk function of genotype effect model involves both the additive and dominance effect while taking into account possible interactions between genes expressed in terms of interaction between their additive and dominance components, while the additive effect model only involves the additive effect and possible interactions. The additive effect model contains less parameters than the genotype effect model. In applications, the additive effect models should be used in analyzing data as the first step. If the dominance effect is strong enough to compensate the increase of the number of the parameters in the genotype effect models, one may use the genotype effect models.

The proposed method has several unique advantages. First, the observed genetic information enters the model directly and the LD structure is captured in the regression coefficients. This aspect offers advantages from the practical point of view, the computational burden is less demanding because haplotype phase need not to be estimated. In the cases when LD is moderate, which is the focus of our work, the computational demands can be substantial even with the current state of technology. Furthermore, the risk due to uncertainty associated with the haplotype phase estimation can be avoided. Second, the estimating procedure is based on a pseudolikelihood model, similarly to the method investigated previously, that allows efficient estimation of parameters, models environmental covariates completely nonparametrically, and incorporates information about the probability of disease [3, 11, 12]. In epidemiologic studies, the vector of environmental covariates measured exactly is often, high dimensional, and a good estimate about probability of disease in a population is known. Additionally, the Bayesian formulation of the proposed method allows shrinking parameter estimates towards prior which offers advantage in cases when misclassification is present.

Because of the Bayesian formulation and the need to validate posterior sets obtained using a pseudolikelihood, the proposed method can be highly computationally intensive. Moreover, the validation of pseudolikelihood requires evaluation of ratio of two likelihood functions. For example, in our simulation experiments and data analysis, this part required us to obtain a precise value of ratios similar to exp(3000)/exp(2908). Hence, we employed GNU Multiple Precision Arithmetic Library (http://gmplib.org/).

The form of our pseudolikelihood function is complex and it does not seem feasible to validate a pseudolikelihood function algebraically. Instead, we propose to apply Monahan and Boos method to examine coverage probabilities of posterior sets. If a comprehensive set of scenarios fails to invalidate a pseudolikelihood function, we suppose that the pseudolikelihood is valid. This reasoning may be similar to the conventional hypothesis testing where the null hypothesis is assumed to be true (pseudolikelihood is valid), and the observed data is used to quantify evidence in favor of the alternative hypothesis (pseudolikelihood is not valid). Of course, a strong basis for validity of a pseudolikelihood is needed. We employ the following arguments. Our previous research approach [3, 11, 12] demonstrated validity of this pseudolikelihood in frequentist sense, that is, we have shown that estimation and inferences are correct when this pseudolikelihood is used in place of a real likelihood function. Hence, posterior distribution based on a pseudolikelihood may be invalid only for certain prior distributions. Therefore, to invalidate a pseudolikelihood, one should find a prior distribution for which the posterior is not valid. However, in our setting, the number of possible prior settings is narrow, because what we advocate is the use of symmetry of prior distribution as a way to improve precision of estimation and inference. We are restricting the prior of regression coefficients to be Gaussian and advocate mean zero and large variance. While one can try other priors for other parameters, the number of possible prior settings is still reasonable and it is practically feasible to look at their performance in terms of probability of coverage sets.

While the major motivation of the proposed work is dictated by the need of a symmetric prior on risk coefficients, other types of *a priori* information can enter our model. For example, if *a priori* information about the LD structure is available, it can be modeled in the *a priori* distribution. Furthermore, if misclassification probabilities are not known precisely, one can specify uncertainty associated with values of misclassification.

A major practical advantage of this proposed work is that it allows the model to exploit recent advances in genotyping technology. Specifically, with the recent advances genetic markers become more and more densely typed and multiple markers are likely to be observed in a functional unit of interest. These units of interest may be defined in terms of LD blocks using information available in linkage maps. While in situations when linkage disequilibrium is strong, the haplotype-based analysis is advantageous; in more common scenarios when linkage disequilibrium is moderate, our approach provides advantages.

However, in the context when the number of genetic markers in a functional unit of interest is large our methodology may require model averaging and model selection component. Hence, behavior of this pseudolikelihood needs to be examined in this setting. A practical strategy can be that one starts with screening analysis first to get interesting genetic variants and SNPs using traditional methods which is computationally less demanding. Then, one may apply the proposed approaches for possible gene-environment interactions and further investigations by focusing on these important and interesting genetic variants and SNPs.

## Figures and Tables

**Table 1:** Biases and root mean squared errors (RMSEs) of risk parameters for the naive approach that ignores existence of misclassification and the proposed method in the case when pr(*D* = 1) is known and when it is estimated. The results are based on 500 samples of 1,500 cases and 1,500 controls. Genotype is simulated at the three marker loci with *P*_*M*_*i*__ = 0.25, *i* = 1, 2, 3, with linkage disequilibrium corresponding to Δ_*M*_*i*_*M*_*j*__ = 0.03. The environmental covariate (*X*) is binary and measured with error with misclassification probabilities being 0.20 for exposed and 0.25 for nonexposed subjects. The data is simulated and analyzed under the genotype effect model.

Parameter	True value	Naive analysis		Pseudo-MLE		MCMC	
		Bias	RMSE	Bias	RMSE	Bias	RMSE
*κ*	0.484	0.481	0.231	−0.054	0.020	−0.032	0.013
*β_X_*	0.693	−0.351	0.132	0.014	0.039	0.008	0.021
*β_A_*_1_	0.406	0.257	0.073	−0.011	0.016	−0.003	0.009
*β_A_*_2_	0.789	0.194	0.046	−0.003	0.015	−0.002	0.006
*β_A_*_3_	0.693	0.283	0.089	−0.005	0.016	−0.003	0.008
*β_AX_*_1_	0.916	−0.425	0.193	0.039	0.046	0.017	0.025
*β_AX_*_2_	0.693	−0.317	0.113	0.038	0.041	0.023	0.021
*β_AX_*_3_	1.099	−0.515	0.282	0.039	0.058	0.019	0.032
*β_D_*_1_	0.262	0.299	0.133	0.026	0.152	0.009	0.368
*β_D_*_2_	0.095	0.258	0.105	0.005	0.099	0.003	0.039
*β_D_*_3_	0.693	0.231	0.092	0.018	0.128	0.008	0.087
*β_DX_*_1_	1.099	−0.495	0.326	0.018	0.301	0.006	0.093
*β_DX_*_2_	0.916	−0.413	0.235	0.006	0.208	0.005	0.121
*β_DX_*_3_	1.099	−0.486	0.313	0.023	0.286	0.017	0.138
*P*_*M*_*i*__	0.250	<0.001	<0.001	−0.001	<0.001	<0.001	<0.001
pr(*X* = 1)	0.500			0.003	0.001	<0.001	<0.001
pr(*D* = 1)	0.005			0.003	<0.001	<0.001	<0.001

**Table 2:** Biases and root mean squared errors (RMSEs) of risk parameters obtained based on pseudo-MLE and the proposed MCMC. The results are based on 500 samples of 350 cases and 350 controls. Genotype is simulated at the two marker loci with *P*_*M*_*i*__ = 0.25, *i* = 1, 2. The environmental covariate (*X*) is binary and measured with error misclassification probabilities being 0.20 for exposed and 0.25 for nonexposed subjects. The data is simulated and analyzed under the additive effect model and the LD measure Δ*_M_*_1_*_M_*_2_ = 0.03.

Parameter	True value	Pseudo-MLE		MCMC	
		Bias	RMSE	Bias	RMSE
*β_X_*	1.099	0.035	0.392	0.013	0.236
*β_A_*_1_	0.406	−0.268	1.035	−0.079	0.397
*β_A_*_2_	0.789	−0.319	1.062	−0.085	0.372
*β_A_*_3_	0.693	−0.293	1.043	−0.092	0.365
*β_AX_*_1_	0.916	0.432	1.135	0.103	0.432
*β_AX_*_2_	0.693	0.391	1.047	0.085	0.481
*β_AX_*_3_	1.099	0.293	1.113	0.097	0.427

**Table 3:** Biases and root mean squared errors (RMSEs) of risk parameters obtained based on asymptotic posterior distribution. The results are based on 500 samples of 1,500 cases and 1,500 controls. Genotype is simulated at the two marker loci with *P*_*M*_*i*__ = 0.25, *i* = 1, 2. The environmental covariate (*X*) is binary and measured with error with misclassification probabilities being 0.20 for exposed and 0.25 for nonexposed subjects. The data is simulated and analyzed under the additive effect model and the LD measure Δ*_M_*_1_*_M_*_2_ = 0.03.

Parameter	True value	Bias	Estimated SE	SE
*β_X_*	0.693	0.010	0.032	0.039
*β_A_*_1_	0.406	−0.005	0.012	0.015
*β_A_*_2_	0.789	−0.004	0.011	0.014
*β_A_*_3_	0.693	−0.004	0.016	0.016
*β_AX_*_1_	0.916	0.023	0.045	0.044
*β_AX_*_2_	0.693	0.019	0.061	0.058
*β_AX_*_3_	1.099	0.020	0.052	0.054
*β_D_*_1_	0.262	0.016	0.431	0.410
*β_D_*_2_	0.095	0.009	0.052	0.063
*β_D_*_3_	0.693	0.013	0.099	0.100
*β_DX_*_1_	1.099	0.011	0.013	0.015
*β_DX_*_2_	0.916	0.013	0.025	0.027
*β_DX_*_3_	1.099	0.016	0.027	0.030

**Table 4:** Risk parameter estimates and standard errors in the alcohol dependence data.

Gene, SNP	Estimate of log(OR)	Standard error
NCAM1, rs586903	1.78	0.06
NCAM1, rs2303377	2.58	0.11
NCAM1, rs2156485	1.87	0.07
TTC12, rs7103866	2.21	0.03
TTC12, rs723077	1.92	0.03
TTC12, rs2288159	2.21	0.01
CHRNA3, rs1051730	1.77	0.03
CHRNA3, rs8192475	1.62	0.02
